# *De novo* Portal Vein Thrombosis in Non-Cirrhotic Non-Alcoholic Fatty Liver Disease: A 9-Year Prospective Cohort Study

**DOI:** 10.3389/fmed.2021.650818

**Published:** 2021-04-29

**Authors:** Ahmed Abdel-Razik, Nasser Mousa, Walaa Shabana, Ahmed H. Yassen, Mostafa Abdelsalam, Mohamed M. Wahba, Eman Mohamed Helmy, Ahmed M. Tawfik, Khaled Zalata, Ahmad S. Hasan, Rania Elhelaly, Rasha Elzehery, Aya Ahmed Fathy, Niveen El-Wakeel, Waleed Eldars

**Affiliations:** ^1^Tropical Medicine Department, Faculty of Medicine, Mansoura University, Mansoura, Egypt; ^2^Nephrology and Dialysis Unit, Internal Medicine Department, Faculty of Medicine, Mansoura University, Mansoura, Egypt; ^3^Diagnostic & Interventional Radiology Department, Faculty of Medicine, Mansoura University, Mansoura, Egypt; ^4^Pathology Department, Faculty of Medicine, Mansoura University, Mansoura, Egypt; ^5^Clinical Pathology Department, Faculty of Medicine, Mansoura University, Mansoura, Egypt; ^6^Public Health and Community Department, Faculty of Medicine, Mansoura University, Mansoura, Egypt; ^7^Medical Microbiology and Immunology Department, Faculty of Medicine, Mansoura University, Mansoura, Egypt

**Keywords:** adiponectin, leptin, leptin/adiponectin ratio, NAFLD, portal vein thrombosis, waist circumference

## Abstract

**Background and Aims:** Approximately 30–40% of portal vein thrombosis (PVT) remains of unknown origin. The association between non-alcoholic fatty liver disease (NAFLD) and PVT is a matter of debate. This study aimed to investigate the association between PVT and NAFLD.

**Methods:** We included 94 out of 105 consecutive NAFLD patients in this prospective cohort study in addition to 94 from the healthy control group. We evaluated biochemical, clinical, immunological, and histopathological parameters; waist circumference (WC); leptin; adiponectin; and leptin/adiponectin ratio (LAR) for all participants at baseline and every 3 years thereafter. We described the characteristics of participants at baseline and showed individual WC, LAR, and PVT characteristics. Potential parameters to predict PVT development within 9 years were determined.

**Results:** PVT developed in eight (8.5%) patients, mainly in the portal trunk. Univariate analysis showed three PVT-associated factors: diabetes mellitus (*P* = 0.013), WC (*P* < 0.001), and LAR (*P* = 0.002). After adjusting multiple confounding variables, the multivariate model showed that the only significant variables were WC and LAR. By applying the receiver operating characteristic curve, WC had 98.8% specificity, 87.5% sensitivity, and 0.894 area under the curve (AUC) for prediction of PVT (*P* < 0.001) at cutoff values of > 105 cm. In comparison, LAR had 60.5% specificity, 87.5% sensitivity, and 0.805 AUC for PVT prediction (*P* < 0.001) at cutoff values of >7.5.

**Conclusions:** This study suggests that increased central obesity and LAR were independently associated with PVT development in non-cirrhotic NAFLD patients, and they should be considered risk factors that may participate in PVT multifactorial pathogenesis.

## Introduction

Cirrhosis and malignancy-free portal vein thrombosis (PVT) is a rare disease with serious complications. The acute form may predispose to intestinal ischemia with an up to 50% mortality rate (1). In developed countries, the first etiology of non-cirrhotic portal hypertension is the chronic form (2). Searching for a precipitating factor is mandatory where systemic or local factors are observed in 70 and 30% of the PVT etiologies, respectively (3, 4). The causes of PVT are multifactorial in ~30% of cases (5). However, 30–40% of cases remain of unknown etiology (6). The pathogenesis of PVT may include one or more risk factors of Virchow's triad: hypercoagulable state, reduced blood flow in the portal vein, or injury in the vascular endothelium (7, 8).

In the United States and other parts of the world, non-alcoholic fatty liver disease (NAFLD) is the most common liver disorder, with a histopathological spectrum extending from hepatic steatosis to non-alcoholic steatohepatitis (NASH). However, not all patients progress through the full hepatological spectrum of NAFLD (9). It is the most common etiology of elevated liver enzymes and is diagnosed after ruling out other steatosis causes, especially alcohol abuse and infectious hepatitis. NAFLD is the hepatic manifestation of metabolic syndrome (10). Dramatically, the prevalence of obesity and metabolic syndrome has been increased and is considered a public health challenge (11). Waist–hip ratio and body mass index (BMI) are used to evaluate obesity. Increased waist circumference (WC) is a cornerstone in the association between venous thromboembolism (VTE) and obesity (12–14). According to visceral fat distribution, abdominal obesity is the leading risk factor for VTE. In cirrhotic patients, obesity is an independent risk factor for PVT (15). PVT's significance is recently starting to be comprehended entirely because its presence may be associated with complications negatively affecting the quality of life, clinical deterioration, hepatic decompensation, and increased mortality after liver transplantation (16).

This work aims to study the clinical and biochemical markers of non-cirrhotic NAFLD patients to identify high-risk factors for PVT development in patients attending our department.

## Materials and Methods

### Patient Characteristics

According to autopsy findings, the incidence of PVT is ~1% (17), and we hypothesized that this might reach higher levels (11–12%) in our patients within the follow-up period. We calculated the sample size at power 99% with a type 1 error = 0.01; the minimal number of cases in each group was 91.

This longitudinal prospective cohort study was conducted on 105 consecutive NAFLD patients admitted to the Mansoura University Hospital, Tropical Medicine Department (Mansoura, Egypt) from April 2006 to December 2018. All patients underwent a complete clinical examination, biochemical appraisal, radiological evaluation, histopathological characteristics, and history taking. All patients with persistently increased liver enzymes in the absence of any etiologies of elevated aminotransferases and hepatosteatosis on ultrasonography were enrolled.

The control group also included 94 healthy sex- and age-matched individuals (female/male = 64/30) who are clinically, biochemically, and radiologically free of NAFLD criteria and have no risk factors predisposing to it. We used hepatic steatosis index and NAFLD-liver fat score to characterize NAFLD's presence or absence in the control group (18, 19).

The patients were enrolled after the exclusion of all possible etiologies of chronic and acute hepatic disorders. Patients who were diagnosed with autoimmune hepatitis, viral hepatitis, human immunodeficiency virus (HIV), hemochromatosis, Wilson's disease, primary biliary cirrhosis, α1-antitrypsin deficiency, sclerosing cholangitis, alcoholic liver disease, NASH in liver biopsy, schistosomiasis (Bilharziasis), or malignancy (especially HCC) were excluded. Patients with collagen vascular diseases, bone marrow depression, chronic renal diseases, acute or chronic pancreatitis, septicemia, postoperative abdominal surgery, liver abscess, diverticulitis, personal or family history of deep vein thrombosis, usage of hepatotoxic, NSAIDs, anticoagulant or antiplatelet therapy, and oral contraceptive drugs were also excluded from this study.

Patients who had patent paraumbilical vein, reversed portal blood flow, inherited coagulation abnormalities, myeloproliferative disorders, peripheral vascular disease, heart failure, hemostatic disorders, clinically overt hyperthyroidism/hypothyroidism, Budd–Chiari syndrome, or liver transplantation were ruled out of this work.

The participants were followed up for 9 years and were assessed at baseline and every 3 years after that by BMI, liver function tests, complete blood count (CBC), C-reactive protein (CRP), activated partial thromboplastin time (APTT), fibrinogen, lipid profile, HOMA-IR, WC, leptin, adiponectin, leptin/adiponectin ratio (LAR), and abdominal Doppler US.

Based on PVT development during follow-up, we evaluated the patients' demographic and clinical characteristics at baseline for their prognostic significance. We analyzed gender, age, BMI, liver function tests, APTT, CRP, HOMA-IR, protein C, protein S, Antithrombin III, serum homocysteine, D-dimer, anti-nuclear antibody (ANA), anti-cardiolipin IgG antibodies (ACA-IgG), anti-double-stranded DNA (anti-dsDNA), WC, leptin, adiponectin, and LAR between PVT and non-PVT patients.

We carried an etiological evaluation for each patient, including antiphospholipid syndrome, myeloproliferative disorders, protein C, protein S and antithrombin III deficiency, paroxysmal nocturnal hemoglobinuria, serum homocysteine, connective-tissue diseases, local risk factors, prothrombin gene mutation, JAK2 V617F mutation, and factor V Leiden especially for patients who developed PVT during the follow-up period (3).

### Clinical Assessments

We interviewed the patients to assess smoking habits, gender, age, and replacement/hormonal therapy and calculated BMI as weight in kilograms divided by height in meters squared. Health experts use the WC, a measurement taken around the abdomen at the umbilicus level, to screen patients for possible weight-related health problems. WC appears to be a better indicator than the waist-to-hip ratio and BMI. WC measurement is convenient, and it is more strongly associated with cardiovascular risk factors and intra-abdominal fat content (20). Measurement of WC was done according to Ma et al. (21), who reported that WC-midabdominal (WC-mid) is a better measurement to define central obesity than WC-Iliac crest (WC-IC). WC-mid was more closely related to metabolic variables and abdominal visceral fat area and had better results for predicting and identifying metabolic diseases.

### Biochemical Assessments

Serum fibrinogen levels, APTT, and prothrombin time (PT) were measured using kits from Siemens Healthcare Diagnostic Inc. (Erlangen, Germany). Serum triglycerides (TG) and cholesterol were measured using kits from Spinreact [Sant Esteve De Bas (GI), Spain]. CRP was measured on COBAS c111 Chemistry Analyzer (Roche Diagnostics, Basel, Switzerland) using commercially available reagents. Serum insulin was measured using RayBio Human Insulin ELISA Kit (3607 Parkway Lane, Suite 100 Norcross, GA 30092). The homeostasis model assessment (HOMA) method (22) was assessed as follows: Insulin resistance (HOMA-IR) = fasting glucose (mmol/L) × fasting insulin (μU/ml)/22.5. Anti-dsDNA, ANA, and ACA-IgG were measured by enzyme-linked immunosorbent assay (ELISA) from Orgentec Diagnostic (Mainz, Germany). Protein C and protein S antigens were measured using ELISA from Corgenix, Inc. (11575 Main Street, SUlte400, Broomfield, CO 80020 USA). Antithrombin III concentration was measured by rate nephelometry from Beckman Coulter, Inc., (Kraemer Blvd., Brea, CA 92821, USA). Semiquantitative evaluation of D-dimer was measured by Tulip Diagnostics Private Ltd. (Alto Santacruz, India). Human Coagulation Factor VIII ELISA Kit was made by MyBioSource, Inc. (San Diego, CA 92195-3308 USA). Plasminogen Activator Inhibitor-1 (PAI-1) ELISA kit was made by R&D Systems (614 McKinley Place NE Minneapolis, MN 55413). Serum homocysteine was measured using an ELISA kit made by DRG International Inc. (841 Mountain Avenue, Springfield, New Jersey 07081, USA). Serum leptin was measured by ELISA from Ray Biotech (Norcross, Georgia, USA). Serum adiponectin was measured by ELISA from Société de Pharmacologie et d'Immunologie–BIO (SPI-BIO) (Montigny le Bretonneux, France). The leptin/adiponectin ratio (LAR) was calculated.

### Radiological Assessments

In all participants, abdominal ultrasound (using standardized criteria) was carried out using a convex probe with a 3.5–5 MHz frequency (SonoAce X6 Ultrasound System; Medison Electronics, Seoul, Korea).

After overnight fasting, examination using a color Doppler ultrasonography was performed on all participants using the same previously mentioned machine. The portal vein (PV) was assessed according to the current guidelines that diminish interobserver variability to non-significant levels (23). The portal flow velocity and PV diameter were measured automatically by the instrument. Doppler examinations were performed on all participants by two different experienced sonographers blinded to the biochemical and clinical data.

The presence of a filling defect of color Doppler US or a grayscale endoluminal material in the main trunk of the PV or its branches may suspect PVT. Computed tomography portal angiography was done for all cases with PVT, which differentiated the complete and partial obstructive thrombosis and extension to the superior mesenteric vein (SMV) and the splenic vein precisely.

PVT was categorized according to Yerdel et al. into four grades. Grade 1: partial PVT (<50% of the lumen) with or without minimal extension into the SMV. Grade 2: >50% occlusion with or without minimal extension into the SMV. Grade 3: complete thrombosis of both proximal SMV and PV with open distal SMV. Grade 4: complete thrombosis of the proximal and distal SMV and PV (24).

### Histopathology Assessments

Patients underwent ultrasound-guided liver biopsy between May 2006 to September 2008, and then they were followed up regularly until December 2018. Hepatic tissues were evaluated by a single pathologist (blinded to clinical and biochemical data), and the histopathological diagnosis was assessed utilizing hematoxylin and eosin stain and Masson trichrome stains of formalin-fixed, paraffin-embedded hepatic tissue. NASH was diagnosed according to Brunt's criteria (25). Based on the NAFLD scoring system, histopathological characteristics were proposed and categorized by the National Institute of Diabetes and Digestive and Kidney Diseases NASH Clinical Research Network (26). NAFLD Activity Score (NAS) provides a composite score based on lobular inflammation, degree of steatosis, and hepatocyte ballooning. A score of 0–2 is simple steatosis, a 3 or 4 is borderline NASH, and ≥5 is likely to represent NASH (26).

NAFLD fibrosis score (NFS) (27) and Fibrosis-4 (FIB-4) score (28) have been developed as alternatives to liver biopsy and have been used as non-invasive tools to detect progression of fibrosis during the entire follow-up period.

### Therapeutic Assessment

Patients received treatment for NAFLD according to the guidelines, and they were all controlled regarding DM and other comorbidities.

All patients with PVT received enoxaparin at a dose of 1 mg/kg SC/12 h for a total duration of 6 months. The schedule and dose of low-molecular-weight heparin (LMWH) varied according to the clinical status and patient's general condition. If thrombosis recurred or remained, the treatment with LMWH could be resumed or continued, or other anticoagulation agents could be used (29).

We carried out a follow-up CT angiography for response evaluations every 90 days or when clinically pertinent events happened. Therapeutic responsiveness was classified as follows: complete recanalization (complete disappearance of the intravenous thrombus), partial recanalization (decreased but remaining thrombus at >25% based on the cross-section of the vessel), stable disease (no change or reduction of the thrombus volume of <25% of the cross-section of the vessel), or progressive status (increased thrombus size). The overall recanalization rate was defined as the sum of the fraction of patients who had partial or complete recanalization (29).

We evaluated and analyzed data on therapeutic outcomes, details of the anticoagulation therapy, and biochemical data at the beginning and the end of the anticoagulation therapy, and overall outcomes were collected through a survey of medical records and the possible adverse events during the LMWH therapy. Based on the International Society on Thrombosis and Hemostasis (ISTH) definition published in 2005, major hemorrhagic adverse events were also defined (30). All the patients had upper gastrointestinal (GI) endoscopy (Olympus GIF-Q200, Olympus Optical Co. Ltd., Tokyo, Japan) before starting anticoagulant treatment.

### Ethics

This study's protocol was approved by the Mansoura Faculty of Medicine Institutional Research Board “MFM-IRB” (Approval no. R/17.11.84), and all methods were performed following relevant guidelines and regulations. Informed consent was obtained from all participants.

### Statistical Analysis

The results were achieved by the Social Package of Statistical Science (SPSS) software version 20 (SPSS Inc., Chicago, IL, USA). Quantitative and non-normally distributed continuous data are described as mean ± SD and (interquartile) range, respectively. We used the Kolmogorov–Smirnov test to determine the compatibility of normally distributed data, Student *t*-test for normally distributed data, Mann–Whitney *U*-test for non-normally distributed continuous data, and Chi-square test for categorical data. Spearman's correlation analysis was carried out between PVT development and other variables. A scatter plot matrix showing bivariate relationships between combinations of different variables was carried out. At univariate analysis, variables with a *P* < 0.05 were enrolled in the multivariable Cox regression analysis. Univariate and multivariable Cox regression models were assessed to identify the independent variables that can be utilized to predict PVT. The receiver operating characteristic curve (ROC) and area under the curve (AUC) were performed, and the best cutoff values were calculated to predict the development of PVT. A two-tailed *P* < 0.05 was considered significant.

## Results

### Patient Characteristics

A total of 105 patients who met the inclusion criteria were enrolled in this study. Of these, 94 patients have completed the study. Baseline biochemical, clinical, and demographic parameters of the enrolled patients are listed in Table 1, and histopathological characteristics and non-invasive fibrosis scores (NFS and FIB-4) are shown in Table 2. Patients showed a statistically significant increase in BMI, ALT, AST, ALP, WC, TG, fibrinogen, antithrombin III, PAI-1, homocysteine, D-dimer, CRP, HOMA-IR, leptin, and LAR compared to that of the control group (all *P* < 0.05). Also, patients showed a statistically significant decrease in adiponectin levels compared to that of the control group (*P* < 0.001).

**Table 1:** Biochemical, clinical, and demographic characteristics of enrolled patients and control group at the baseline of the study.

**Parameters**	**Patient group** ** (*n* = 94)**	**Control group** ** (*n* = 94)**	***P*-value**
Age (years)	48 (39–58)	48 (39–57)	0.772
Sex (female/male)	65/29	64/30	0.96
Smoking habits			
Current smoker	42 (45)	40 (43)	0.783
Ex-smoker	18 (19)	19 (20)	0.863
Never smoked	34 (36)	35 (37)	0.887
Hypertension	23 (24)	–	–
DM	32 (34)	–	–
BMI (kg/m^2^)	27.3 ± 3.5	23.8 ± 1.1	<0.001
ALT (U/L)	68 (47–95)	29 (20–36)	<0.001
AST (U/L)	54 (46–75)	21 (18–32)	<0.001
γ-GT (IU/L)	39 (30–54)	34 (30–50)	0.216
ALP (IU/L)	96 (83–110)	89 (78–101)	0.024
Albumin (g/dl)	4.4 ± 1.3	4.3 ± 1.1	0.57
Bilirubin (mg/dl)	1.15 ± 0.3	1 ± 0.2	0.18
PT (s)	12 ± 0.8	11.8 ± 0.7	0.07
APTT (s)	35.1 ± 5.1	34 ± 4.8	0.13
Serum creatinine (mg/dl)	0.8 (0.6–1.39)	0.74 (0.6–1.2)	0.502
Waist circumference (cm)	91.6 ± 4.3	82.2 ± 1.9	<0.001
Waist circumference, cm men >102, women >88	54 (57)	–	-
Serum total cholesterol (mg/dl)	197 ± 62	185 ± 22	0.079
Serum triglyceride (mg/dl)	139 (90–184)	93 (80–113)	<0.001
Fibrinogen levels (mg/dl)	269 (227–347)	182 (141–214)	<0.001
Protein C (IU/dl)	82.5 ± 8	80.5 ± 7.8	0.084
Protein S (IU/dl)	85.9 ± 7.5	83.8 ± 7.2	0.052
Antithrombin III	82.5 ± 5.4	79.1 ± 5.1	<0.001
Factor VIII (ng/ml)	101.7 ± 9.5	99.5 ± 8.8	0.101
PAI-1 (ng/ml)	22.4 ± 3.7	10.5 ± 2.3	<0.001
Homocysteine (μmol/L)	13.3 ± 0.7	12.9 ± 0.6	<0.001
D-dimer (ng/ml)	656 (370–826)	425 (244–624)	<0.001
ANA positive (*n*) (positive ≥1.2)	3	–	–
Anti-dsDNA positive (*n*) (positive >20 IU/ml)	0	–	–
ACA-IgG positive (*n*) (positive ≥10 U/ml)	0	–	–
CRP (mg/L)	66 (40–79)	9.0 (4.7–11.6)	<0.001
HOMA-IR	3.2 (1.4–4.1)	1.8 (1.2–2.4)	<0.001
Leptin (ng/ml)	130.2 ± 8.1	95.4 ± 5.3	<0.001
Adiponectin (μg/ml)	20.56 ± 1.2	25.6 ± 1.8	<0.001
LAR	6.3 ± 0.43	3.8 ± 0.51	<0.001
Portal how velocity (cm/s)	23.8 ± 4.4	25 ± 4.5	0.066

*The statistics presented are means ± SD, N (%), or median and interquartile range. DM, diabetes mellitus; BMI, basal metabolic index; ALT, alanine aminotransferase; AST, aspartate aminotransferase; γ-GT, γ-glutamyl transpeptidase; ALP, alkaline phosphatase; PT, prothrombin time; APTT, activated partial thromboplastin time; ANA, anti-nuclear antibody; Anti-dsDNA, anti-double-strand DNA; ACA-IgG, anti-cardiolipin IgG antibody; CRP, C-reactive protein; HOMA-IR, homeostasis model assessment-insulin resistance; LAR, leptin/adiponectin ratio; NAS, non-alcoholic fatty liver disease activity score; NASH, non-alcoholic steatohepatitis*.

**Table 2:** Histopathological characteristics and fibrosis scores of enrolled patients at the baseline of the study.

**Histopathological characteristics**	
Degree of inflammation	
No (minimal)/mild/moderate/severe	0/12/52/30
Grades of steatosis	
Non/Grade 1/Grade 2/Grade 3	0/24/43/27
Ballooning	
None/Few/Many	0/63/31
Stages of fibrosis	
0/1/2/3/4	3/9/50/32/0
NAS (non-alcoholic fatty liver disease activity score)	
0–2 (simple steatosis)	21
3–4 (borderline NASH)	29
5–8 (NASH)	44
**Non-invasive fibrosis scores**	
NAFLD fibrosis score (NFS)	0.18 (−1.45 to 0.37)
Fibrosis-4 (FIB-4)	2 (1.38–3.36)

The participants were followed up every 3 years for 9 years after the initial assessment at baseline by hematological and biochemical blood tests and the abdominal Doppler US.

**Figure 1:**
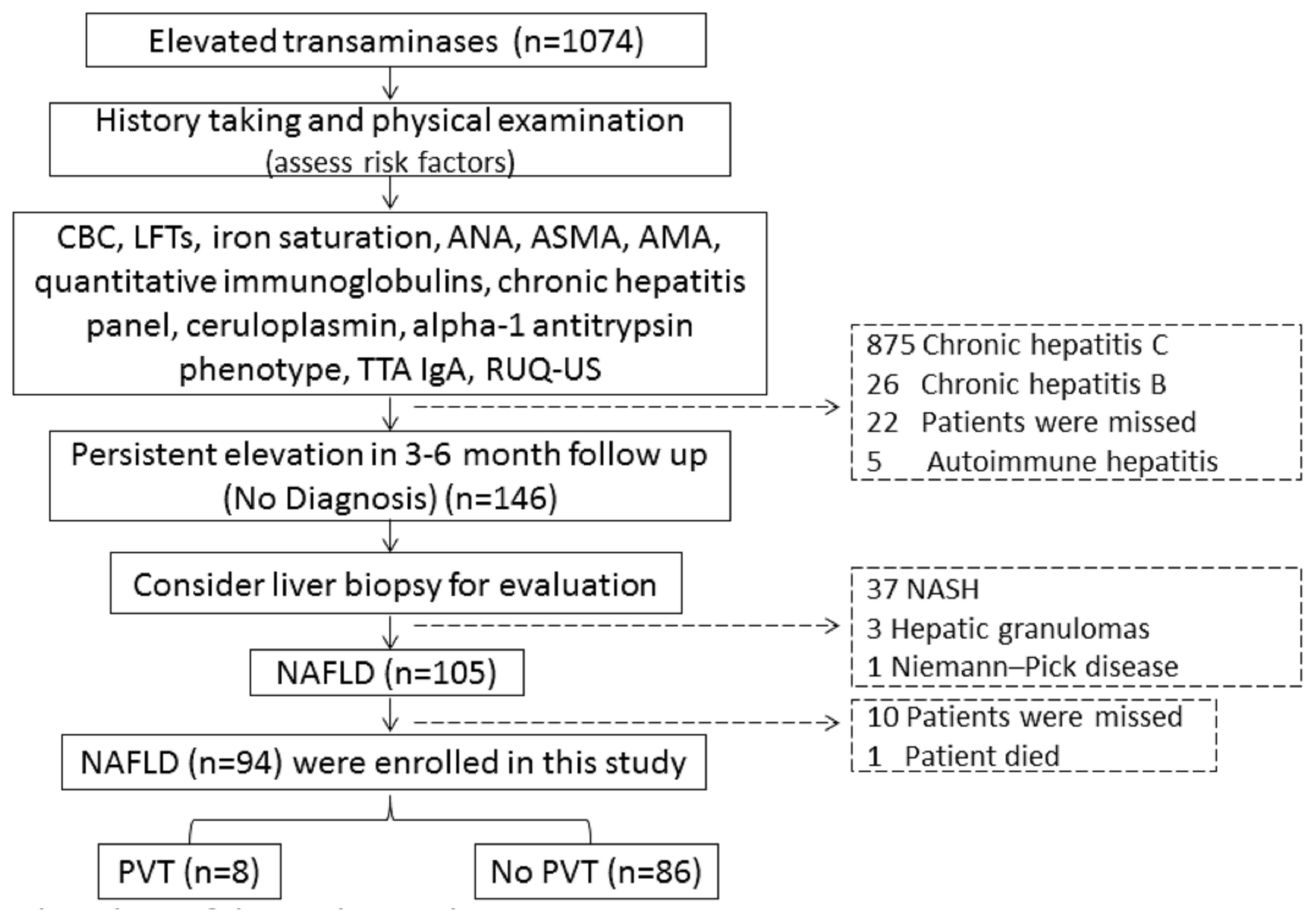
Flow chart of the study population. CBC, complete blood picture; LFTs, liver function tests, ANA, antinuclear antibody; ASMA, anti-smooth muscle antibody; AMA, antimitochondrial antibodies; TTA lgA, tissue transglutaminase lgA; RUQ-US, right upper quadrant abdominal-ultrasonography; NASH, non-alcoholic steatohepatitis; NAFLD, non-alcoholic fatty liver diseases; PVT, portal vein thrombosis.

**Figure 2:**
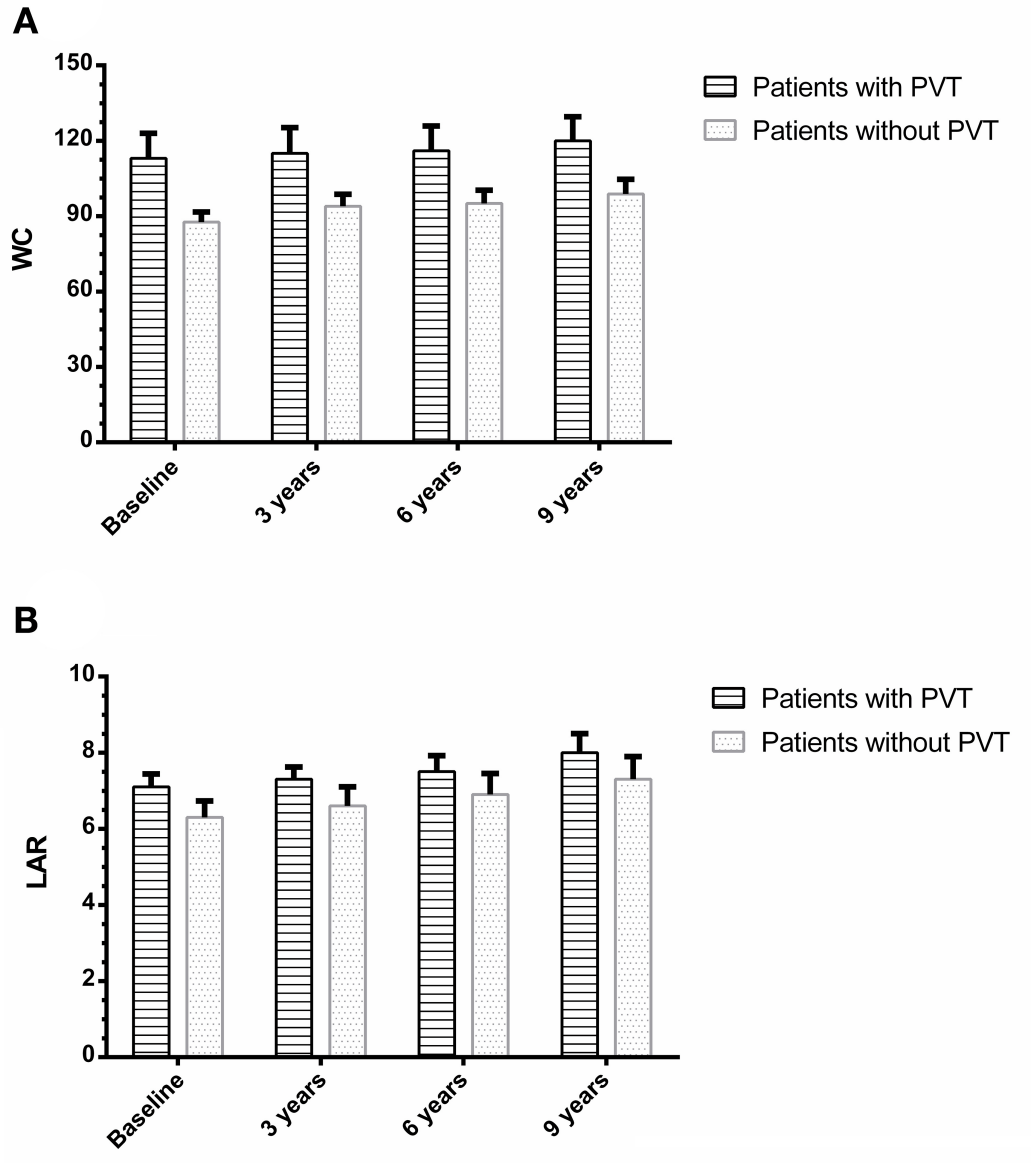
Waist circumference **(A)** and leptin/adiponectin ratio **(B)** changes in patients with and without portal vein thrombosis.

Ninety-four of the 105 patients were included to complete this work; 10 patients were missed during the follow-up period. One patient died in a motor car accident. Out of all the patients examined during the follow-up period (*n* = 94), eight patients (8.5%) developed *de-novo* PVT, as shown in Figure 1. Individual WC, LAR, and non-invasive fibrosis scores (NFS and FIB-4) of patients with PVT are shown in Table 3, while WC and LAR changes in patients without PVT are displayed in Figure 2. None of the control group (*n* = 94) showed any abnormality regarding PVT diagnostic criteria when they were enrolled in the study or later on till the end of the follow-up period.

**Table 3:** WC, LAR, and fibrosis scores in patients who developed PVT within 9 years of observation.

**Patient No**.	**WC**				**LAR**			
	**Baseline**	**3 years**	**6 years**	**9 years**	**Baseline**	**3 years**	**6 years**	**9 years**
4	90	91	93	98	6.8	7.1	7.6	8.6
11	115	117	118	121	6.9	7	7.1	7.4
27	115	116	117	122	7.3	7.5	7.9	8.4
32	114	116	118	120	7.4	7.6	7.8	8.1
49	119	120	121	125	7.5	7.7	8.1	8.5
63	117	119	120	125	6.9	7.1	7.2	7.6
74	123	125	126	130	6.5	6.8	6.9	7.6
85	111	113	115	118	7.1	7.3	7.5	8
**Patients with PVT** ** (*****n*** **=** **8)**	**113** **±** **10**	**115** **±** **10.2**	**116** **±** **9.9**	**120** **±** **9.6**	**7.1** **±** **0.34**	**7.3** **±** **0.32**	**7.5** **±** **0.42**	**8** **±** **0.5**
		*P1* = 0.7	*P2* = 0.85	*P3* = 0.43		*P1 =* 0.25	*P2 =* 0.3	*P3 =* 0.048
				*P4* = 0.18				*P4* < 0.001
**Patients without PVT** ** (*****n*** **=** **86)**	**87.6** **±** **4.1**	**93.9** **±** **4.8**	**95.1** **±** **5.2**	**98.8** **±** **5.86**	**6.3** **±** **0.43**	**6.6** **±** **0.5**	**6.9** **±** **0.55**	**7.3** **±** **0.6**
		P1* < 0.001	P2^*^ = 0.12	P3* < 0.001		P1^*^ < 0.001	P2^*^ < 0.001	P3^*^ < 0.001
				P4^*^ < 0.001				P4^*^ < 0.001
**Non-invasive fibrosis scores**								
	Baseline	3 years	6 years	9 years				
NFS in *PVT*	**0.22** ** (−1.45–0.55)**	**0.23** ** (−1.47–0.56)**	**0.24** ** (−1.49–0.59)**	**0.27** ** (−1.51–0.62)**	*P1, P2, P3*, and *P4* > 0.05			
FIB-4 in *PVT*	**2.45** ** (1.23–3.33)**	**2.47** ** (1.26–3.36)**	**2.51** ** (1.28–3.39)**	**2.52** ** (1.31–3.42)**	*P1, P2, P3* and *P4* > 0.05			
NFS in *non-PVT*	**0.175** ** (−1.45–0.37)**	**0.182** ** (−1.41–0.38)**	**0.184** ** (−1.38–0.39)**	**0.191** ** (−1.35–0.41)**	P1*, P2*, P3*, and P4* > 0.05			
FIB-4 in *non-PVT*	**1.88** ** (1.4–3.36)**	**1.92** ** (1.4–3.38)**	**1.94** ** (1.5–3.41)**	**1.95** ** (1.5–3.41)**	P1*, P2*, P3*, and P4* > 0.05			

*WC, Waist circumference; LAR, leptin/adiponectin ratio; PVT, portal vein thrombosis; NFS, NAFLD fibrosis score; FIB-4, Fibrosis-4*.

P1: 3 years vs. baseline in patients with PVT. P1^*^: 3 years vs. baseline in patients without PVT.

P2: 6 years vs. 3 years in patients with PVT. P2^*^: 6 years vs. 3 years in patients without PVT.

P3: 9 years vs. 6 years in patients with PVT. P3^*^: 9 years vs. 6 years in patients without PVT.

P4: 9 years vs. baseline in patients with PVT. P4^*^: 9 years vs. baseline in patients without PVT.

The control group did not show any statistically significant changes in LAR levels between the baseline and 3-year follow up, 6-year follow-up, or at the end of study (3.8 ± 0.51 vs. 3.85 ± 0.52, *P* = 0.61; 3.8 ± 0.51 vs. 3.9 ± 0.52, *P* = 0.18; and 3.8 ± 0.51 vs. 3.95 ± 0.54, *P* = 0.052) correspondingly. There were no statistically significant changes in WC between the baseline and 3-year follow up, 6-year follow-up, or at the end of study (82.2 ± 1.9 vs. 81.9 ± 1.8, *P* = 0.27; 82.2 ± 1.9 vs. 82.5 ± 2, *P* = 0.29; and 82.2 ± 1.9 vs. 82.6 ± 2.1, *P* = 0.17), respectively. There were no statistically significant changes in all variables between the baseline and at the end of study (all *P* > 0.05) (data not shown).

The main clinical complaints in patients who develop PVT were abdominal pain (seven patients, 87.5%) and asymptomatic (one patient, 12.5%). However, the signs varied from one case to another, irrespective of PVT sites.

### Correlation Between WC and LAR With Clinical, Biochemical, and Histopathological Patterns of the Studied Patients

Actually, in Spearman correlation analysis, there were significant positive correlations between WC and age, serum fibrinogen, CRP, degree of steatosis, grades of inflammation, fibrosis scores, ballooning, and NAFLD activity score (NAS) (*rho* = 0.61, *P* = 0.001; *rho* = 0.57, *P* = 0.019; *rho* = 0.78, *P* < 0.001; *rho* = 0.72, *P* < 0.001; *rho* = 0.69, *P* < 0.001; *rho* = 0.76, *P* < 0.001; *rho* = 0.78, *P* < 0.001; and *rho* = 0.73, *P* < 0.001, respectively).

Moreover, in Spearman correlation analysis, there were significant positive correlations between LAR values and age, serum fibrinogen, serum homocysteine, CRP, degree of steatosis, grades of inflammation, fibrosis scores, ballooning, and NAS (*rho* = 0.63, *P* = 0.001; *rho* = 0.59, *P* = 0.016; *rho* = 0.55, *P* < 0.001; *rho* = 0.79, *P* < 0.001; *rho* = 0.74, *P* < 0.001; *rho* = 0.71, *P* < 0.001; *rho* = 0.77, *P* < 0.001; *rho* = 0.79, *P* < 0.001; and *rho* = 0.75, *P* < 0.001, respectively).

Also, there was a significant positive correlation between WC and LAR (*rho* = 0.71, *P* < 0.001).

### Univariate and Multivariable Cox Regression Models Predicting PVT Within 9 Years Follow-Up

The biochemical, demographic, and clinical parameters of patients with and without PVT and characteristics of PVT are listed in Supplementary Tables 1, 2.

Regarding sex, age, hypertension, BMI, smoking habits, ALT, AST, γ-GT, ALP, serum albumin, serum bilirubin, PT, APTT, serum creatinine, serum triglyceride, fibrinogen levels, serum CRP, HOMA-IR, protein C, protein S, Antithrombin III, Factor VIII, PAI-1, serum homocysteine, D-dimer, ANA, ACA-IgG, and anti-dsDNA, the difference was not significant between the two groups (all *P* > 0.05).

Univariate Cox regression analysis revealed that diabetes mellitus, increased WC, and LAR are significant predictors of PVT (all *P* < 0.05) in Table 4.

**Table 4:** The risk of portal vein thrombosis development in the univariate and multivariable Cox regression models in the studied patients.

**Parameters**	**Univariate Cox regression**		**Multivariable Cox regression**	
	**HR (95% CI)**	***P*-value**	**HR (95% CI)**	***P*-value**
DM	6.34 (1.29–31.19)	0.013	1.23 (0.15–10.32)	0.85
Waist circumference	1.16 (1.09–1.24)	<0.001	1.18 (1.09–1.28)	<0.001
LAR	7.44 (1.93–28.87)	0.002	8.04 (1.62–39.9)	0.011
NFS	0.97 (0.45–2.09)	0.93	–	-
FIB-4	1.03 (0.52–2.050)	0.92	–	-

*DM, diabetes mellitus; LAR, leptin/adiponectin ratio; CI, confidence interval; HR, hazard ratio; NFS, NAFLD fibrosis score; FIB-4, Fibrosis-4*.

After adjusting multiple confounders, the multivariable Cox regression analysis model was re-evaluated using the formerly described baseline parameters related to PVT's occurrence during the 9 years follow-up period. This analysis revealed that WC and LAR are independent factors associated with PVT development (Table 4).

**Figure 3:**
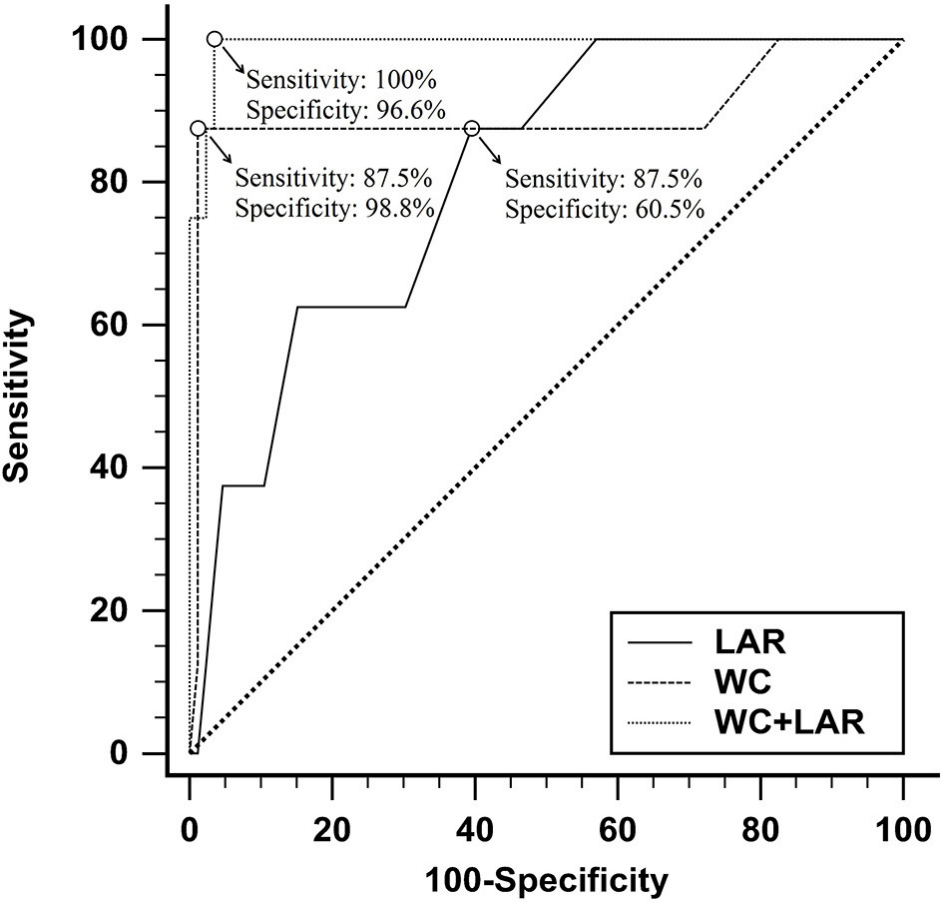
The receiver operating characteristic curves of waist circumference and leptin/adiponectin ratio in predicting portal vein thrombosis in non-cirrhotic NAFLD.

Using the ROC curve analysis, at a cutoff value of >105 cm and 7.5, WC and LAR had (98.8%, 60.5%) specificity, (87.5%, 87.5%) sensitivity, (0.894, 0.805) AUC, (87.5%, 17.1%) positive predictive value (PPV), and (98.8%, 98.1%) negative predictive value (NPV) correspondingly for prediction of PVT (*P* < 0.001). Combined WC and LAR were identified as the best discriminating markers in the prediction of PVT with 96.6% specificity, 100% sensitivity, 72.7% PPV, 100% NPV, and 0.993 AUC (*P* < 0.001), as shown in Figure 3.

### Therapeutic Findings

Enoxaparin was administered to all patients who developed PVT. All patients had a complete response after 6 months of follow-up. The median duration of the anticoagulation therapy was 3.31 ± 1.6 months (range, 1–6 months). The average interval from the onset of therapy to the first CT was 87 days. The overall recanalization rate was 100% (eight patients). Complete recanalization was achieved in seven patients (87.5%), and partial recanalization was reported in one patient (12.5%). No one showed any criteria for stable and/or progressive disease during LMWH therapy. In all patients showing complete and/or partial recanalization, none of them showed PVT relapse or progression after the end of anticoagulation therapy.

At the time of the analysis, all patients had completed the treatment with LMWH; 8 patients (100%) completed a 6-month treatment regimen. No clinically relevant bleeding was reported.

All included laboratory values and metabolic parameters are not significantly changed before and after the LMWH treatment (data not shown). All patients that reached the endpoint are still followed regularly in our outpatient clinic till the present day.

In addition, there is no bleeding or major complications reported during the LMWH therapy.

## Discussion

The relationship between a hypercoagulable state and NAFLD is a perpetually extending field of research. NAFLD is the hepatic manifestation of a metabolic syndrome often associated with thrombosis and hypercoagulability with NAFLD's natural history (9).

The current study reported that *de novo* PVT incidence within NAFLD patients during a 9-year follow-up was 8.5%.

Generally, the possible explanation may be that NAFLD's continuous and chronic inflammation leads to lipid-based oxidative injury, necrosis, and apoptosis (10, 31–33). This clarifies the stimulation of the coagulation cascade and the resultant hypercoagulable condition because procoagulant levels of factor VIII and plasminogen activator inhibitor-1 (PAI-1) have been increased, while anticoagulant levels of protein C are diminished in the late stage of NAFLD patients (34). Contrary to expectations, this study did not find a significant difference between procoagulant levels of factor VIII and PAI-1 as well as the anticoagulant level of protein C in patients with or without PVT.

In this study, multivariable Cox regression analysis revealed that increased WC and LAR were independently associated with PVT development in non-cirrhotic NAFLD patients.

It is well-known that one of the biological variations noted in metabolic syndrome is an increase in coagulation factors. Many factors known to be related to thrombosis and fat mass risk could be included, such as serum leptin or PAI-1 (35). Besides that, adipose tissue may produce an excess amount of proteins that behave in a paracrine, autocrine, or endocrine manner. The lipid accumulation is correlated with the production of inflammatory cytokines and increased macrophage infiltration of adipose tissue. In obese individuals, the inflammatory changes present in adipose tissue may induce liver inflammation and hemostatic abnormalities.

Moreover, overweight is a well-known deep vein thrombosis risk factor (12, 13). Increased WC may be considered a landmark of metabolic syndrome and obesity (36). Abdominal obesity was implicated in VTE and coronary heart disease (37, 38) through hypercoagulability via the decreased fibrinolytic process and elevated levels of factor VIII and fibrinogen (39, 40). Abdominal obesity often has a pro-inflammatory state, characterized by elevated acute-phase reactants such as fibrinogen and CRP and a pro-thrombotic condition due to increased levels of PAI-1, clotting factors, and fibrinogen (41, 42). Both pro-thrombotic and pro-inflammatory states are closely linked to VTE development. These results clinically support the hypercoagulable state of abdominal obesity related to PVT in NAFLD patients. This observation is in accordance with Bureau et al., who reported that central obesity is related to PVT and could become one of the critical risk variables for gastrointestinal thrombosis (43).

Our findings declared that increased LAR was an independent factor associated with the development of PVT in NAFLD. As we know, in diabetes mellitus, obesity, and other metabolic syndromes, the concurrence of hyperleptinemia and hypoadiponectinemia is observed. Different studies highlighted the association between LAR and cardiovascular disease markers, including pulse wave velocity and carotid intima-media thickness (44, 45). LAR correlated with HOMA-IR, WC, BMI, and TG better than any adipokine (44). In NAFLD patients, higher levels of LAR were observed. As adiponectin and leptin generally show opposite variations, leptin appeared to upregulate different vascular inflammation mediators such as ROS, IL-2, IL-6, TNF-α, MCP-1, TGF-α, and Th1-type cytokines from peripheral blood mononuclear cells and endothelial cells (46–48). Experimentally, leptin prompted increments of cellular adhesion molecules and tissue factor expression in human coronary endothelial cells, though NF-κB leads to increased leukocyte adhesion and procoagulant activity (49). These results in patients with metabolic syndrome exhibit a strong relationship with increased platelet activity and circulating leptin (50–52). The leptin receptor is expressed on endothelial cells and platelets and could enhance the formation of thrombus by inhibiting vasodilatation, stimulating platelet activation, and increasing oxidative stress (53).

It is well-known that diabetes, obesity, and lower adiponectin levels are related to the stimulation of an inflammatory signaling cascade, prompting the early development of atherosclerosis in the metabolic syndrome (54). Adiponectin suppresses the endothelium's vascular inflammatory effect to TNF-α-induced stimulation of NF-κB and enhanced expression of adhesion molecules, intercellular adhesion molecules, vascular cell adhesion molecules, and endothelial selectin. Experimentally, reversed microvascular inflammatory changes may be induced by adiponectin replacement therapy (55). Vascular effects induced by pro-inflammatory cytokines like TNF-α and different interactions with adipokines (adiponectin) greatly enhance vascular thromboembolism (56). Bureau et al. observed that PVT is associated with increased abdominal obesity and could get to be distinctly one of the critical risk factors for gastrointestinal thrombosis (43). According to these findings, we hypothesized that the LAR ratio plays a pivotal role in developing PVT in our patients.

LMWH has no impact on coagulation tests' consequences and offers the advantages of dosages proportional to body weight. Therefore, it is easy to prescribe and does not require monitoring. The risk of bleeding is the primary concern related to anticoagulation therapy for PVT. In this study, the overall recanalization rate was 100%, and all treated patients did not reveal any bleeding or significant complications. All patients that reached the endpoint are still followed up regularly.

Central obesity and NAFLD are two sides of a single coin. In addition to endothelial dysfunction and liver inflammation, it is challenging to speculate each variable's role in PVT development. However, the authors' conviction is that central obesity may play a fundamental role in PVT development through hormonal imbalance, cytokine production, and hepatic affection.

To the best of our knowledge, this study is the first to measure the incidence of PVT in patients with non-cirrhotic NAFLD.

This study had several limitations. First, it is a single-center study. Second, the follow-up period was only 9 years. Third, using non-invasive techniques like NFS and FIB-4 score cannot replace the liver biopsy, but we thought it would be unethical to subject our patients to a technique as invasive as the biopsy for the second time because nothing changed, which was indicative for the biopsy during the study period. The findings of this study have several important implications for future practice. Multicenter research is usually conducted to enroll larger numbers of participants and thus improve the validity and generalizability of the study findings.

Finally, we recommend that early distinguishing of risk variables, management, and prevention of the metabolic syndrome, including lifestyle modifications and treatment for adjusting the syndrome components, are real difficulties. The point is to avert obesity, type 2 diabetes, hyperlipidemia, cardiovascular disease, and PVT.

In conclusion, this work suggests that increased WC and LAR in NAFLD patients are associated independently with PVT development. We propose that these variables may be considered risk factors for PVT and may participate in PVT's multifactorial pathogenesis.

## Data Availability Statement

The data that support the findings of this study have restrictions and so are not publicly available. Data are however available from the authors upon reasonable request.

## Ethics Statement

The studies involving human participants were reviewed and approved by Mansoura Faculty of Medicine Institutional Research Board MFM-IRB (Approval no. R/17.11.84). The patients/participants provided their written informed consent to participate in this study.

## Author Contributions

AA-R, NM, WS, and AY designed the study and/or contributed to the concept and performed the statistical analysis. MA, MW, EH, and AT interpreted data critically, contributed to data acquisition, and revised the manuscript. AH drafted the manuscript. KZ and RElz critically recruited and followed up with patients and revised the manuscript. RElh analyzed, acquired, and interpreted data and revised the manuscript. AF, NE-W, and WE contributed to analyzing and interpreting data and performed the statistical analysis. All authors approved the final version of the article.

## Conflict of Interest

The authors declare that the research was conducted in the absence of any commercial or financial relationships that could be construed as a potential conflict of interest.

## Abbreviations

NAFLD: non-alcoholic fatty liver disease
PVT: portal vein thrombosis
VTE: venous thromboembolism
LAR: leptin/adiponectin ratio
WC: waist circumference
HCC: Hepatocellular carcinoma
HOMA-IR: Homeostatic model assessment—insulin resistance
BMI: body mass index.

## References

[1] Spaander VM van Buuren HR Janssen HL. The management of non-cirrhotic non malignant portal vein thrombosis and concurrent portal hypertension in adults. Aliment Pharmacol Ther. (2007) 26(Suppl. 2):203–9. 10.1111/j.1365-2036.2007.03488.x 18081663

[2] Valla D-C Condat B Lebrec D. Spectrum of portal vein thrombosis in the West. J Gastroenterol Hepatol. (2002) 17:S224–7. 10.1046/j.1440-1746.17.s3.4.x 12472940

[3] Plessier A Darwish-Murad S Hernandez-Guerra M Consigny Y Fabris F Trebicka J. Acute portal vein thrombosis unrelated to cirrhosis: a prospective multicenter follow-up study. Hepatology. (2010) 51:210–8. 10.1002/hep.23259 19821530

[4] Janssen HL Wijnhoud A Haagsma EB van Uum SH van Nieuwkerk CM Adang RP. Extrahepatic portal vein thrombosis: aetiology and determinants of survival. Gut. (2001) 49:720–4. 10.1136/gut.49.5.720 11600478 PMC1728504

[5] Denninger MH Chaït Y Casadevall N Hillaire S Guillin MC Bezeaud A. Cause of portal or hepatic venous thrombosis in adults: the role of multiple concurrent factors. Hepatology. (2000) 31:587–91. 10.1002/hep.510310307 10706547

[6] Turon F Cervantes F Colomer D Baiges A Hernández-Gea V Garcia-Pagán JC. Role of calreticulin mutations in the etiological diagnosis of splanchnic vein thrombosis. J Hepatol. (2015) 62:72–4. 10.1016/j.jhep.2014.08.032 25173966

[7] Chawla YK Bodh V. Portal vein thrombosis. J Clin Exp Hepatol. (2015) 5:22–40. 10.1016/j.jceh.2014.12.008 25941431 PMC4415192

[8] Cruz-Ramón V Chinchilla-López P Ramírez-Pérez O Aguilar-Olivos NE Alva-López LF Fajardo-Ordoñez E. Thrombosis of the portal venous system in cirrhotic vs. non-cirrhotic patients. Ann Hepatol. (2018) 17:476–81 10.5604/01.3001.0011.7392 29735798

[9] Brunt EM. Non-alcoholic steatohepatitis: pathologic features and differential diagnosis. Semin Diagn Pathol. (2005) 22:330–8 10.1053/j.semdp.2006.04.002 16939061

[10] Argo CK Caldwell SH. Epidemiology and natural history of non-alcoholic steatohepatitis. Clin Liver Dis. (2009) 13:511–31. 10.1016/j.cld.2009.07.005 19818302

[11] Ng M Fleming T Robinson M Thomson B Graetz N Margono C. Global, regional, and national prevalence of overweight and obesity in children and adults during 1980-2013: a systematic analysis for the Global Burden of Disease Study 2013. Lancet. (2014) 384:766–81. 10.1016/S0140-6736(14)60460-8 24880830 PMC4624264

[12] Severinsen MT Kristensen SR Johnsen SP Dethlefsen C Tjønneland A Overvad K. Anthropometry, body fat, and venous thromboembolism: a Danish follow-up study. Circulation. (2009) 120:1850–7. 10.1161/CIRCULATIONAHA.109.863241 19858417

[13] Steffen LM Cushman M Peacock JM Heckbert SR Jacobs DR Jr Rosamond WD. Metabolic syndrome and risk of venous thromboembolism: longitudinal investigation of thromboembolism etiology. J Thromb Haemost. (2009) 7:746–51. 10.1111/j.1538-7836.2009.03295.x 19175496 PMC2810102

[14] Di Minno MN Tufano A Rusolillo A Di Minno G Tarantino G. High prevalence of non-alcoholic fatty liver in patients with idiopathic venous thromboembolism. World J Gastroenterol. (2010) 16:6119–22. 10.3748/wjg.v16.i48.6119 21182227 PMC3012581

[15] Ayala R Grande S Bustelos R Ribera C García-Sesma A Jimenez C. Obesity is an independent risk factor for pre-transplant portal vein thrombosis in liver recipients. BMC Gastroenterol. (2012) 12:114 10.1186/1471-230X-12-114 22909075 PMC3502589

[16] Rodríguez-Castro KI Porte RJ Nadal E Germani G Burra P Senzolo M. Management of nonneoplastic portal vein thrombosis in the setting of liver transplantation: a systematic review. Transplantation. (2012) 94:1145–53. 10.1097/TP.0b013e31826e8e53 23128996

[17] Ogren M Bergqvist D Björck M Acosta S Eriksson H Sternby NH. Portal vein thrombosis: prevalence, patient characteristics and lifetime risk: a population study based on 23,796 consecutive autopsies. World J Gastroenterol. (2006) 12:2115–9. 10.3748/wjg.v12.i13.2115 16610067 PMC4087695

[18] Lee JH Kim D Kim HJ Lee CH Yang JI Kim W. Hepatic steatosis index: a simple screening tool reflecting non-alcoholic fatty liver disease. Dig Liver Dis. (2010) 42:503–8. 10.1016/j.dld.2009.08.002 19766548

[19] Kotronen A Peltonen M Hakkarainen A Sevastianova K Bergholm R Johansson LM. Prediction of non-alcoholic fatty liver disease and liver fat using metabolic and genetic factors. Gastroenterology. (2009) 137:865–72. 10.1053/j.gastro.2009.06.005 19524579

[20] Pouliot MC Després JP Lemieux S Moorjani S Bouchard C Tremblay A. Waist circumference and abdominal sagittal diameter: best simple anthropometric indexes of abdominal visceral adipose tissue accumulation and related cardiovascular risk in men and women. Am J Cardiol. (1994) 73:460–8. 10.1016/0002-9149(94)90676-9 8141087

[21] Ma WY Yang CY Shih SR Hsieh HJ Hung CS Chiu FC. Measurement of Waist Circumference: midabdominal or iliac crest? Diabetes Care. (2013) 36:1660–6. 10.2337/dc12-1452 23275359 PMC3661855

[22] Matthews DR Hosker JP Rudenski AS Naylor BA Treacher DF Turner RC. Homeostasis model assessment: insulin resistance and beta-cell function from fasting plasma glucose and insulin concentrations in man. Diabetologia. (1985) 28:412–9. 10.1007/BF00280883 3899825

[23] Sabbà C Merkel C Zoli M Ferraioli G Gaiani S Sacerdoti D. Interobserver and interequipment variability of echo-Doppler examination of the portal vein: effect of a cooperative training program. Hepatology. (1995) 21:428–33. 10.1002/hep.1840210225 7843716

[24] Yerdel MA Gunson B Mirza D Karayalçin K Olliff S Buckels J. Portal vein thrombosis in adults undergoing liver transplantation: risk factors, screening, management, and outcome. Transplantation. (2000) 69:1873–81. 10.1097/00007890-200005150-00023 10830225

[25] Brunt EM Janney CG Di Bisceglie AM Neuschwander-Tetri BA Bacon BR. Non-alcoholic steatohepatitis: a proposal for grading and staging the histological lesions. Am J Gastroenterol. (1999) 94:2467–74. 10.1111/j.1572-0241.1999.01377.x 10484010

[26] Kleiner DE Brunt EM Van Natta M Behling C Contos MJ Cummings OW. Nonalcoholic Steatohepatitis Clinical Research Network. design and validation of a histological scoring system for non-alcoholic fatty liver disease. Hepatology. (2005) 41:1313–21. 10.1002/hep.20701 15915461

[27] Angulo P Hui JM Marchesini G Bugianesi E George J Farrell GC. The NAFLD fibrosis score: a non-invasive system that identifies liver fibrosis in patients with NAFLD. Hepatology. (2007) 45:846–54. 10.1002/hep.21496 17393509

[28] Petroff D Bätz O Jedrysiak K Kramer J Berg T Wiegand J. Fibrosis-4 (FIB-4) score at the primary care level: an analysis of over 160 000 blood samples. Gut. (2021) 70:219–21. 10.1136/gutjnl-2020-320995 32245907

[29] Kwon J Koh Y Yu SJ Yoon JH. Low molecular weight heparin treatment for portal vein thrombosis in liver cirrhosis: Efficacy and the risk of hemorrhagic complications. Thromb Res. (2018) 163:71–6. 10.1016/j.thromres.2018.01.032 29407630

[30] Schulman S Kearon C Subcommittee on Control of Anticoagulation of the Scientific and Standardization Committee of the International Society on Thrombosis and Haemostasis. Definition of major bleeding in clinical investigations of antihemostatic medicinal products in non-surgical patients. J Thromb Haemost. (2005) 3:692–4. 10.1111/j.1538-7836.2005.01204.x 15842354

[31] Targher G Bertolini L Rodella S Lippi G Franchini M Zoppini G. NASH predicts plasma inflammatory biomarkers independently of visceral fat in men. Obesity. (2008) 16:1394–9. 10.1038/oby.2008.64 18369343

[32] Targher G Zoppini G Moghetti P Day CP. Disorders of coagulation and hemostasis in abdominal obesity: emerging role of fatty liver. Semin Thromb Hemost. (2010) 36:41–8. 10.1055/s-0030-1248723 20391295

[33] Cigolini M Targher G Agostino G Tonoli M Muggeo M De Sandre G. Liver steatosis and its relation to plasma haemostatic factors in apparently healthy men—role of the metabolic syndrome. Thromb Haemost. (1996) 76:69–73. 10.1055/s-0038-1650524 8819254

[34] Tripodi A Fracanzani AL Primignani M Chantarangkul V Clerici M Mannucci PM. Procoagulant imbalance in patients with non-alcoholic fatty liver disease. J Hepatol. (2014) 61:148–54. 10.1016/j.jhep.2014.03.013 24657400

[35] Hamsten A Wiman B de Faire U Blombäck M. Increased plasma levels of a rapid inhibitor of tissue plasminogen activator in young survivors of myocardial infarction. N Engl J Med. (1985) 313:1557–63. 10.1056/NEJM198512193132501 3934538

[36] Reilly MP Rader DJ. The metabolic syndrome: more than the sum of its parts? Circulation. (2003) 108:1546–51. 10.1161/01.CIR.0000088846.10655.E0 14517150

[37] Hansson PO Eriksson H Welin L Svardsudd K Wilhelmsen L. Smoking and abdominal obesity: risk factors for venous thromboembolism among middle-aged men: “the study of men born in 1913”. Arch Intern Med. (1999) 159:1886–90. 10.1001/archinte.159.16.1886 10493318

[38] Lakka HM Laaksonen DE Lakka TA Niskanen LK Kumpusalo E Tuomilehto J. The metabolic syndrome and total and cardiovascular disease mortality in middle aged men. JAMA. (2002) 288:2709–16. 10.1001/jama.288.21.2709 12460094

[39] Yudkin JS Juhan-Vague I Hawe E Humphries SE di Minno G Margaglione M. Low-grade inflammation may play a role in the etiology of the metabolic syndrome in patients with coronary heart disease: the HIFMECH study. Metabolism. (2004) 53:852–7. 10.1016/j.metabol.2004.02.004 15254876

[40] Ridker PM Buring JE Cook NR Rifai N. C-reactive protein, the metabolic syndrome, and risk of incident cardiovascular events: an 8- year follow-up of 14 719 initially healthy American women. Circulation. (2003) 107:391–7. 10.1161/01.CIR.0000055014.62083.05 12551861

[41] Imperatore G Riccardi G Iovine C Rivellese AA Vaccaro O. Plasma fibrinogen: a new factor of the metabolic syndrome. a population-based study. Diabetes Care. (1998) 21:649–54. 10.2337/diacare.21.4.649 9571358

[42] Woodward M Lowe GD Rumley A Tunstall-Pedoe H Philippou H Lane DA. Epidemiology of coagulation factors, inhibitors and activation markers: The Third Glasgow MONICA Survey. II. relationships to cardiovascular risk factors and prevalent cardiovascular disease. Br J Haematol. (1997) 97:785–97. 10.1046/j.1365-2141.1997.1232935.x 9217177

[43] Bureau C Laurent J Robic MA Christol C Guillaume M Ruidavets JB. Central obesity is associated with non-cirrhotic portal vein thrombosis. J Hepatol. (2016) 64:427–32. 10.1016/j.jhep.2015.08.024 26334577

[44] Norata GD Raselli S Grigore L Garlaschelli K Dozio E Magni P. leptin: adiponectin ratio is an independent predictor of intima media thickness of the common carotid artery. Stroke. (2007) 38:2844–6. 10.1161/STROKEAHA.107.485540 17823381

[45] Satoh N Naruse M Usui T Tagami T Suganami T Yamada K. Leptin-to-adiponectin ratio as a potential atherogenic index in obese type 2 diabetic patients. Diabetes Care. (2004) 27:2488–90. 10.2337/diacare.27.10.2488 15451921

[46] Avogaro A de Kreutzenberg SV. Mechanisms of endothelial dysfunction in obesity. Clinica Chimica Acta. (2005) 360:9–26. 10.1016/j.cccn.2005.04.020 15982646

[47] Loffreda S Yang SQ Lin HZ Karp CL Brengman ML Wang DJ. leptin regulates pro-inflammatory immune responses. FASEB J. (1998) 12:57–65. 10.1096/fasebj.12.1.57 9438411

[48] S'anchez-Margalet V Mart'in-Romero C Santos-Alvarez J Goberna R Najib S Gonzalez-Yanes C. Role of leptin as an immunomodulator of blood mononuclear cells:mechanisms of action. Clin Exp Immunol. (2003) 133:11–19. 10.1046/j.1365-2249.2003.02190.x 12823272 PMC1808745

[49] Hsuchou H Kastin AJ Tu H Joan Abbott N Couraud PO Pan W. Role of astrocytic leptin receptor subtypes on leptin permeation across hCMEC/D3 human brain endothelial cells. J Neurochem. (2010) 115:1288–98. 10.1111/j.1471-4159.2010.07028.x 20977476 PMC2972375

[50] Thøgersen AM Söderberg S Jansson JH Dahlén G Boman K Nilsson TK. Interactions between fibrinolysis, lipoproteins and leptin related to a first myocardial infarction. Eur J Cardiovasc Prev Rehabil. (2004) 11:33–40. 10.1097/01.hjr.0000116824.84388.a2 15167204

[51] Chu N-F Spiegelman D Hotamisligil GS Rifai N Stampfer M Rimm EB. Plasma insulin, leptin, and soluble TNF receptors levels in relation to obesity-related atherogenic and thrombogenic cardiovascular disease risk factors among men. Atherosclerosis. (2001) 157:495–503. 10.1016/S0021-9150(00)00755-3 11472752

[52] Małyszko J Wołczy'nski S Małyszko J My'sliwiec M. Leptin correlates with some hemostatic parameters in CAPD patients. Nephron. (2002) 92:721–4. 10.1159/000064074 12372966

[53] Schäfer K Konstantinides S. Mechanisms linking leptin to arterial and venous thrombosis: potential pharmacological targets. Curr Pharm Des. (2014) 20:635–40. 10.2174/13816128113199990021 23688015

[54] Weyer C Funahashi T Tanaka S Hotta K Matsuzawa Y Pratley RE. Hypoadiponectinemia in obesity and type 2 diabetes: close association with insulin resistance and hyperinsulinemia. J Clin Endocrinol Metab. (2001) 86:1930–5. 10.1210/jcem.86.5.7463 11344187

[55] Ouchi N Kihara S Arita Y Okamoto Y Maeda K Kuriyama H. Adiponectin, an adipocytederived plasma protein, inhibits endothelial NF-B signaling through a cAMP-dependent pathway. Circulation. (2000) 102:1296–301. 10.1161/01.CIR.102.11.1296 10982546

[56] Adya R Tan BK Randeva HS. Differential effects of leptin and adiponectin in endothelial angiogenesis. J Diabetes Res. (2015) 2015:648239. 10.1155/2015/648239 25650072 PMC4310451

