# Supplementary material for: De novo Portal Vein Thrombosis in Non-Cirrhotic Non-Alcoholic Fatty Liver Disease: A 9-Year Prospective Cohort Study
Source: Front Med (Lausanne). 2021 Apr 29;8:650818. doi: 10.3389/fmed.2021.650818 (PMC8117420; doi:10.3389/fmed.2021.650818)
Supplement: Supplementary file 1 [file Data_Sheet_1.PDF]

## Supplementary Material

**Table S1.** Characteristics of PVT in patients within 9 years of observation.

| Patients No. | PVT at 3 years | | PVT at 6 years | | PVT at 9 years | |
|---|---|---|---|---|---|---|
| | No/ Partial /Complete | Site and extension | No/ Partial /Complete | Site and extension | Partial /Complete | Site and extension |
| 4 | No | - | Partial (G1) † | Portal trunk | Partial (G1) † | Portal trunk |
| 11 | No | - | No | - | Partial (G2) ‡ | Rt. portal branch |
| 27 | No | - | No | - | Partial (G1) † | Portal trunk |
| 32 | Partial (G1) † | Portal trunk | Partial (G1) † | Portal trunk | Partial (G1) † | Portal trunk |
| 49 | No | - | No | - | Complete (G3) * | Portal trunk & SMV |
| 63 | No | - | No | - | Partial (G1) † | Portal trunk |
| 74 | No | - | Partial (G1) † | Rt. portal branch | Partial (G1) † | Rt. portal branch |
| 85 | No | - | No | - | Partial (G2) ‡ | Portal trunk |

PVT, portal vein thrombosis; SMV, superior mesenteric vein; G1, grade 1; G2, grade 2; G3, grade 3

† Grade 1

‡ Grade 2

* Grade 3

Grade 1, grade 2 and grade 3 were categorized *according to Yerdel* et al. classification [24].

**Table S2.** Biochemical, clinical, and demographic characteristics of patients without and with PVT during follow-up period.

| Parameters | No PVT (*n*=86) | PVT (*n*=8) | *P* value |
|---|---|---|---|
| Age (years) | 48 (39-58) | 44 (41-56) | 0.791 |
| Sex (female/male) | 60/26 | 5/3 | 0.91 |
| Smoking habits | | | |
| Current smoker | 38 (44.2) | 4 (50) | 0.754 |
| Ex-smoker | 16 (18.6) | 2 (25) | 0.662 |
| Never smoked | 32 (37.2) | 2 (25) | 0.494 |
| Hypertension | 20 (23.3) | 3 (34) | 0.502 |
| DM | 26 (30) | 6 (75) | 0.011 |
| BMI (kg/m2) | 26.7 ± 2.8 | 28.1 ± 3.4 | 0.187 |
| ALT (U/l) | 67 (36-75) | 72 (51-86) | 0.356 |
| AST (U/l) | 54 (39-67) | 54 (46-74) | 0.528 |
| γ-GT (IU/l) | 35 (29-54) | 44 (35-59) | 0.326 |
| ALP (IU/l) | 90 (75-99) | 96 (88-114) | 0.081 |
| Albumin (g/dl) | 4.1±1.3 | 3.9±1.2 | 0.677 |
| Bilirubin (mg/dl) | 1.11±0.19 | 1.17±0.2 | 0.397 |
| PT (Sec.) | 11.7 ± 1.1 | 12.3 ± 1.3 | 0.149 |
| APTT (Sec.) | 35.5 ± 4.9 | 38.2 ± 5.2 | 0.141 |
| Serum creatinine (mg/dl) | 0.8±0.53 | 1.1±0.58 | 0.132 |
| Waist circumference (cm) | 98.8±5.86 | 120±9.58 | <0.001 |
| Waist circumference (cm) men >102, woman >88 | 47 (55) | 7 (87.5) | 0.077 |
| Serum total cholesterol (mg/dl) | 175 ± 52 | 210±69 | 0.08 |
| Serum triglyceride (mg/dl) | 129 (94-180) | 136 (107-200) | 0.538 |
| Fibrinogen levels (mg/dl) | 241 (217-333) | 299 (239-347) | 0.347 |
| Protein C (IU/dL) | 82±7.8 | 87.6±9.1 | 0.06 |
| Protein S (IU/dL) | 85.6±7.8 | 89.4±1.6 | 0.174 |
| Antithrombin | 82.3±4.7 | 85±9.6 | 0.166 |
| Factor VIII (ng/ml) | 101.3±9.5 | 106±9.4 | 0.184 |

| | | | |
|---|---|---|---|
| PAI-1 (ng/ml) | 22.3±3.6 | 23.6±5.1 | 0.349 |
| Homocysteine (μmol/L) | 13.3±0.8 | 13.7±0.8 | 0.18 |
| D-dimer (ng/ml) | 583 (380-835) | 890 (553-920) | 0.17 |
| ANA positive (*n*) (positive ≥1.2) | 2 (2.3) | 1(12.5) | 0.117 |
| Anti-dsDNA positive (*n*) (positive >20 IU/ml) | 0 | 0 | - |
| ACA-IgG positive (*n*) (positive ≥10 U/ml) | 0 | 0 | - |
| CRP (mg/l) | 62 (40 - 78) | 75 (47-83) | 0.403 |
| HOMA-IR | 3.8 (1.2-3.2) | 3.9 (1.9-4.2) | 0.236 |
| Leptin (ng/ml) | 134 ±7.2 | 138.9±1.6 | 0.059 |
| Adiponectin (ug/mL) | 18.1±1 | 17.5±0.8 | 0.103 |
| LAR | 7.4±0.6 | 8±0.5 | 0.007 |
| Portal flow velocity (cm/s) | 24.6±4 | 22.8±3.6 | 0.223 |
| Histopathological characteristics: | | | |
| Degree of inflammation | | | |
| No (minimal) /mild/moderate/severe | 0/11/48/27 | 0/1/4/3 | |
| Grades of steatosis | | | |
| Non/Grade 1/Grade 2/Grade 3 | 0/22/39/25 | 0/2/4/2 | 0.95 |
| Ballooning | | | |
| None/Few/Many | 0/58/28 | 0/5/3 | |
| Stages of fibrosis | | | |
| 0/1/2/3/4 | 2/7/47/30/0 | 1/2/3/2/0 | |
| NAS (nonalcoholic fatty liver disease activity score) | | | |
| 0-2 (simple steatosis) | 19 (22) | 2 (25) | |
| 3-4 (borderline-NASH) | 27 (31) | 2 (25) | |
| 5-8 (NASH) | 40 (47) | 4 (50) | 0.82 |

| | | | |
|---|---|---|---|
| Non-invasive Fibrosis scores | | | |
| NAFLD fibrosis score (NFS) | 2.45 (1.23 -3.33) | 0.22 (-1.45 -0.55) | 0.62 |
| Fibrosis-4 (FIB-4) | 1.88 (1.4 - 3.36) | 2.45 (1.23 -3.33) | 0.95 |

The statistics presented are means ± SD, N (%) or *median* and interquartile range

DM, diabetes mellitus; BMI, basal metabolic index; ALT, alanine aminotransferase; AST, aspartate aminotransferase; γ-GT, γ-glutamyl transpeptidase; ALP, alkaline phosphatase; PT, prothrombin time; APTT, activated partial thromboplastin time; ANA, antinuclear antibody; Anti-dsDNA, anti-double-strand DNA; ACA-IgG, anticardiolipin IgG antibody; CRP, C-reactive protein; HOMA-IR, homeostasis model Assessment-insulin resistance; LAR, leptin/adiponectin ratio.
